# Histological and molecular responses of *Vigna angularis* to *Uromyces vignae* infection

**DOI:** 10.1186/s12870-022-03869-2

**Published:** 2022-10-14

**Authors:** Xiwang Ke, Jie Wang, Xiaodan Xu, Yongxia Guo, Yuhu Zuo, Lihua Yin

**Affiliations:** 1 National Coarse Cereals Engineering Research Center, Heilongjiang Provincial Key Laboratory of Crop-Pest Interaction Biology and Ecological Control, Heilongjiang Bayi Agricultural University, 163319 Daqing, China; 2 Department of Biological Center, Harbin Academy of Agricultural Sciences, 150028 Harbin, China

**Keywords:** Adzuki bean rust, Transcriptome, Pattern-recognition receptors, Pathogenesis related protein

## Abstract

**Background:**

To advance the understanding of adzuki bean (*Vigna angularis*) resistance to infection with the rust-causing fungus *Uromyces vignae* (*Uv*), we comprehensively analyzed histological events and the transcriptome of *Uv-*infected adzuki bean.

**Results:**

Compared with the susceptible cv. Baoqinghong (BQH), the resistant cv. QH1 showed inhibition of uredospore germination and substomatal vesicle development, intense autofluorescence of cells around the infection site, and cell wall deposit formation in response to *Uv* infection. In cv. QH1, gene set enrichment analysis (GSEA) showed enrichment of chitin catabolic processes and responses to biotic stimuli at 24 h post-inoculation (hpi) and cell wall modification and structural constituent of cytoskeleton at 48 hpi. Kyoto Encyclopedia of Genes and Genomes (KEGG) analysis indicated enrichment of WRKY transcription factors (TFs), the calcium binding protein cml, and hydroquinone glucosyltransferase at both 24 and 48 hpi. In total, 1992 and 557 differentially expressed genes (DEGs) were identified at 24 and 48 hpi, respectively. Cell surface pattern-recognition receptors (PRRs), WRKY TFs, defense-associated pathogenesis-related (PR) proteins, and lignin and antimicrobial phenolic compound biosynthesis were significantly induced. Finally, we detected the chitinase (CHI) and phenylalanine ammonia-lyase (PAL) activity were higher in QH1 and increased much earlier than in BQH.

**Conclusion:**

In cv. QH1, cell-surface PRRs rapidly recognize *Uv* invasion and activate the corresponding TFs to increase the transcription of defense-related genes and corresponding enzymatic activities to prevent fungal development and spread in host tissues.

**Supplementary information:**

The online version contains supplementary material available at 10.1186/s12870-022-03869-2.

## Background

Plants have evolved a multilayered immune system to protect themselves from various phytopathogens. In addition to passive mechanisms, plants have evolved at least two lines of active defense mechanisms to detect and cope with diverse biotic attacks [1, 2]. The first line of the defense system is triggered by the recognition of pathogen-associated molecular patterns (PAMPs) via cell-surface-localized pattern recognition receptors (PRRs), leading to PAMPs-triggered immunity (PTI) [1]. PTI plays a prominent role in curtailing pathogens during the early stage of invasion [3]. Following invasion, pathogens, including bacteria, fungi, and oomycetes, can deliver virulence-associated molecules, such as effectors secreted into plant cells or apoplasts, to suppress host immunity [4]. To cope with the virulence of these pathogens, plants activate their second-line immune system, known as effector-triggered immunity (ETI), upon the direct or indirect recognition of effectors by intracellular nucleotide-binding domain leucine-rich repeat-containing receptors (NLRs) [1]. Once activated, both PTI and ETI induce a downstream cascade of similar defenses, such as bursts of reactive oxygen species (ROS), the activation of protein kinase cascades, the induction of stress-related hormone signaling pathways, the modification of cell walls and the activation of pathogenesis-related (PR) gene expression [5, 6].

The fungus *Uromyces vignae* (*Uv*) is the causal agent of adzuki bean (*Vigna angularis*) rust [7]. This pathogen employs an obligate biotrophic infection strategy to invade living leaves. The symptoms first appear as small yellow spots on leaf surfaces, and severe infections result in premature leaf drop. Rust is considered to be the biggest threat to dry bean production, leading to up to 18-100% of crop losses [8]; additionally, this disease has been reported to cause a yield loss increase of 19 kg/ha for every 1% increase in disease severity [9]. As a traditional coarse cereal crop and export crop that contributes to foreign exchange, the annual cultivation area of adzuki bean in China is estimated to be 670,000 ha [10]. In addition, adzuki bean seed can provide a high source of protein, starch, mineral elements, and vitamins with low caloric and fat, so it is used in a variety of foods for at least a billion people [11, 12]. Combined with its broad adaptability and ability to improve the soil condition through nitrogen fixation [13], adzuki beans are highly valuable in the crop rotation and coarse cereal export systems in China. The deployment of genetic resistance is the most effective, environmentally friendly, cost-effective, and long-term strategy to control rust. The application of genetically resistant cultivars depends on the source by which resistance genes are identified. However, previous studies have revealed that little of the adzuki bean germplasm exhibited rust resistance [14]. Additionally, limited information regarding the resistance or defensive genes of adzuki bean has been reported [15, 16].

For rust fungi, the infection initiates with uredospore germination and appressorium formation over a stoma and then colonizes by invading through stomatal openings [17]. In this process, the plant cell walls, surface antimicrobial enzymes and secondary metabolites that constitute barriers are often the first group of obstacles faced by the pathogen [18]. In addition to these constitutive barriers, the invading pathogen can potentially activate rapid gene expression and cell wall reinforcement changes to cope with the fungal colonization [19]. Despite the good understanding of the principal defense responses of plants to fungal pathogens, lesser is known about the factors contributing to the resistance of *V. angularis* to *Uv* infection.

To advance our understanding of adzuki bean resistance to *Uv* infection, a comparative study of the infection process of *Uv* on leaves of varieties with different resistances was performed by fluorescence microscopy. To explore the genes that potentially play important roles in regulating defense responses in adzuki beans, time-resolved transcriptomes of the resistant variety were analyzed at different *Uv* infection stages.

## Results

### Uredospores germination and infection on resistant variety leaves were inhibited

**Fig. 1:**
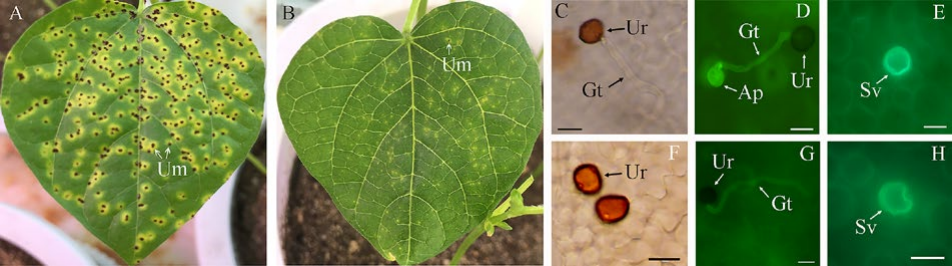
Symptoms and frequency of uredospore germination, infection and Sv deformity on different cultivars. A and B, symptoms on the leaf of the susceptible cv. BQH and resistant cv. QH1 at 10 days post-inoculation. C-E, indicate the uredospore development on susceptible cv. BQH at 24 h post-inoculation (hpi). C, showed the uredospore (Ur) germination with germ tube (Gt), bar = 20 μm. D, showed the appressorium (Ap) formed on stomata, bar = 20 μm. E, showed the normal circular substomatal vesicle (Sv), bar = 10 μm. F-H, showed the uredospore development on resistant cv. QH1 at 24 hpi. F, ungerminated uredospore, bar = 20 μm. G, germinated uredospore without appressorium formation, bar = 20 μm. H, showed the deformity Sv, bar = 10 μm

The *Uv* infection severity on the susceptible adzuki bean variety Baoqinghong (BQH) and the resistant variety QH1 were significantly different. On leaves of BQH, obvious chlorosis lesions were observed at 5 days post-inoculation (dpi), while leaves of QH1 were symptomless. By 8 dpi, large numbers of uredium were observed on BQH, but sporulation was negligible on QH1. Until 10 dpi, in BQH, more uredium was produced, and its diameter increased (Fig. 1 A). In contrast, rarely uredium was observed on the leaf surfaces of QH1 at 10 dpi (Fig. 1B). Microscopically, on the BQH leaves at 24 h post-inoculation (hpi), over 85% (n = 120) of uredospore had generated germ tubes (Fig. 1 C) and appressoria on stoma (Fig. 1D), and over 90% (n = 30) of the appressoria had produced substomatal vesicles (Sv), marking a successful invasion of *Uv* (Fig. 1E). Meanwhile, the QH1 plants showed obvious resistance phenotypes, including a low uredospore germination frequency (56.7%, n = 100, Fig. 1 F) and infection ratio (65.0%, n = 30,) (Fig. 1G). Moreover, a higher frequency of Sv deformities in QH1 (65.7%, n = 30, Fig. 1 H) was also observed. Overall, uredospore germination, stoma penetration and Sv development were significantly reduced during *Uv* infection in the resistant variety QH1 compared to the susceptible variety BQH.

### Infection observation by fluorescence microscopy

**Fig. 2:**
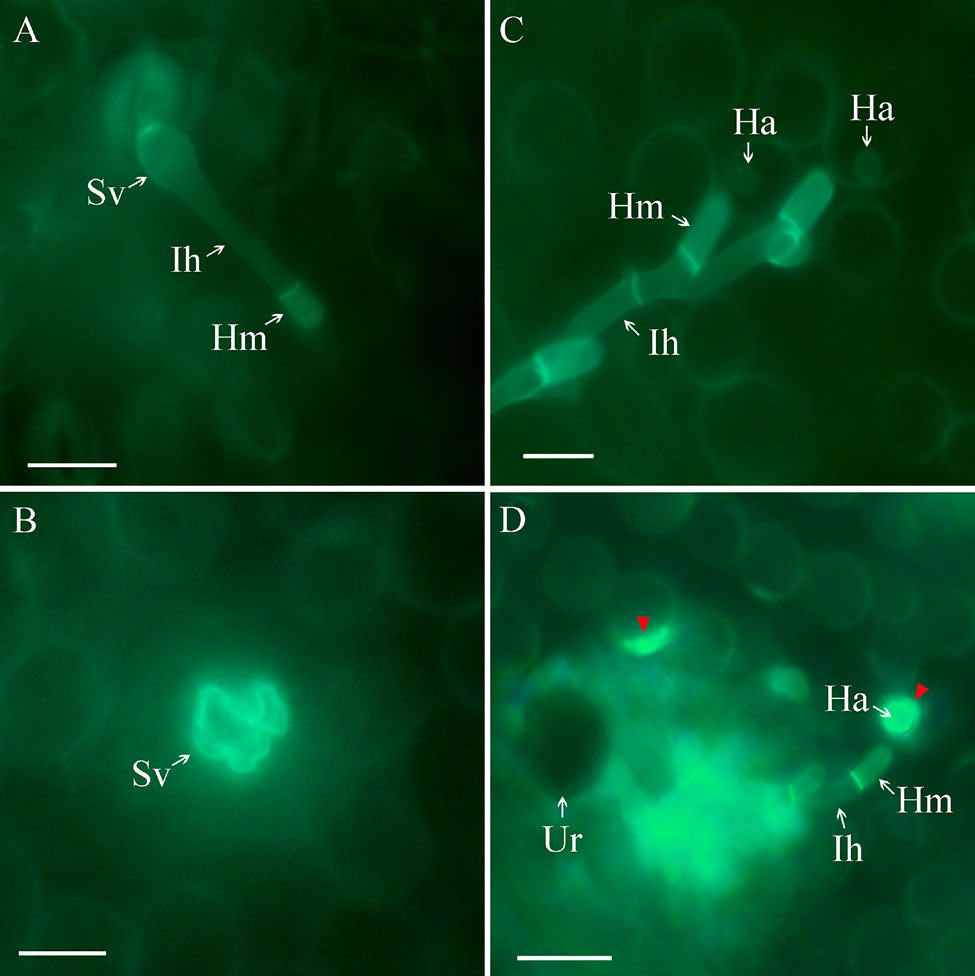
Adzuki bean leaves of different cultivars infected by *U. vignae* (*Uv*) were examined under an epifluorescence microscope after being stained with Calcofluor. (A), infection hyphae (Ih) and Haustoria mother cells (Hm) were generated from substomatal vesicles (Sv) in the susceptible variety (BQH) at 24 hpi. (B), deformed Sv without Ih or Hm developed in the resistant variety (QH1) at 24 hpi. (C), Ih formed branches and produced intracellular haustoria (Ha) in BQH plants at 48 hpi, and the host cells showed no obvious reactions despite the fungal invasion. (D), autofluorescence was observed in the periphery of the Ha (red arrows) and mesophyll cells at the infection sites. The scale bar represents 20 μm

After entering the substomatal cavity, the infection hyphae (Ih) developed from Sv, and a haustoria mother cell (Hm) was formed at the tip of the Ih in BQH at 24 hpi (Fig. 2 A). However, in QH1, most of the Sv could not develop Ih (Fig. 2B) due to the high malformation rate. After the formation of Hm, haustoria (Ha) were generated in plant cells, and Ih branched to spread in the BQH tissue at 48 hpi (Fig. 2 C). The BQH mesophyll cells penetrated by *Uv* remained normal and healthy. In contrast, in the resistant variety, QH1, although a few infectious hyphae breached the cell wall and form haustoria, the haustoria formed by *Uv* in QH1 were encased in callose-like deposits that exhibited strong autofluorescence (Fig. 2D, red arrows). In addition, at the Ih and Hm contact sites, the mesophyll cells showed intense bright fluorescence (Fig. 2D).

### Identification of differentially expressed genes (DEGs) related to *Uv* resistance

**Fig. 3:**
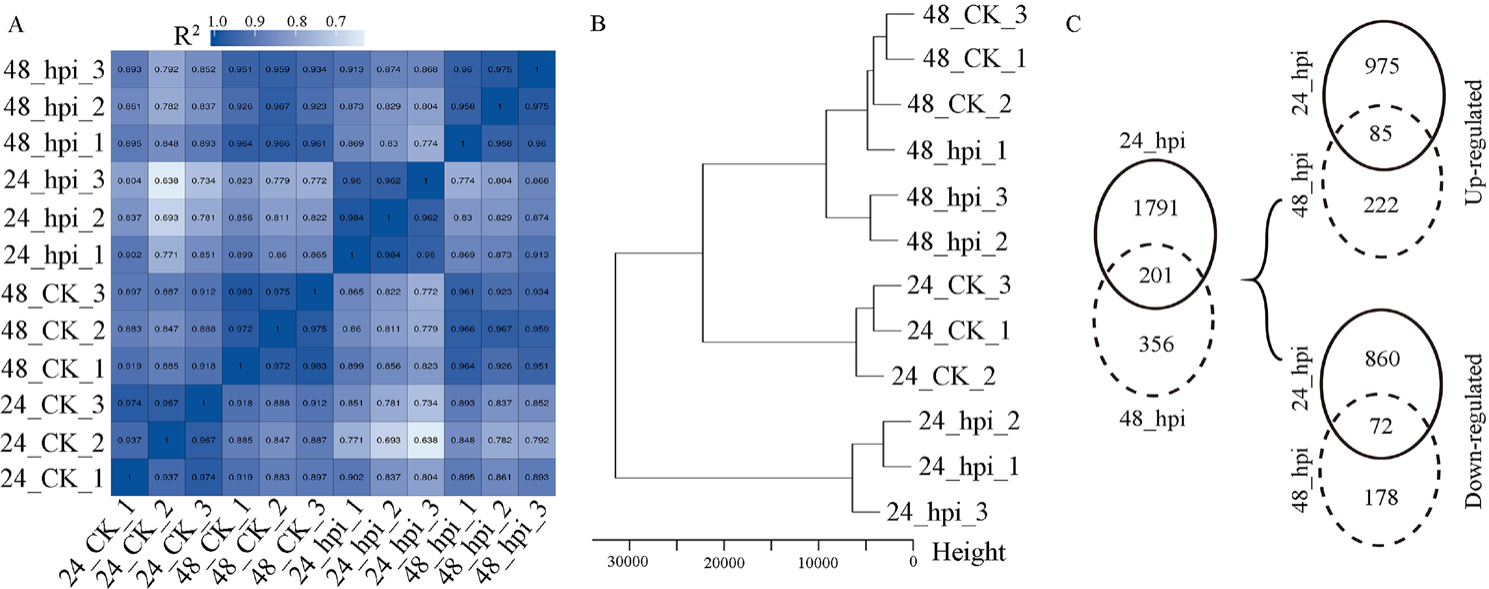
Gene expression correlations among samples derived in the differentially expressed genes (DEGs) analysis. (A), heatmap of the Pearson correlation coefficients derived for the triplicates representing all samples. (B), hierarchical cluster analysis of all samples. The terms 24_CK_1–24_CK_3 represent the different replicates of the control sample at 24 h post-inoculation with sterile water; a similar naming strategy was applied for the 48_CK samples. The terms 24_hpi_1–24_hpi_3 represent the different replicates of the sample at 24 h post-inoculation with *Uv*; a similar naming strategy was applied for the 48_hpi samples. (C), Venn diagram of the DEGs derived in response to the fungal infection at 24 and 48 hpi compared with the mock samples

To identify the genes in the resistant cv. QH1 that are involved in *Uv* resistance, gene expression profiling of the *Uv*-infected leaves at 24- and 48-hours post-inoculation (hpi) was performed via RNA sequencing. A total of 110 million single-end RNA-seq clean reads were obtained (for an average of 9.2 million reads per sample), and these reads were mapped to the *V. angularis* genome. Over 87% of the reads mapped uniquely to the *V. angularis* genome (Additional file 1: Table S1), and a total of 24,710 genes were detected with different expression levels. To assess the repeatability among different samples, a Pearson’s correlation coefficient (R^2^) analysis based on the fragments per kilobase of exon model per million mapped fragments (FPKM) of all detected genes was conducted. The results showed that the R^2^ values derived among the treatment triplicates were greater than 0.93, but those derived among the different treatments were less than 0.9 or even below 0.7 (Fig. 3 A). Additionally, the hierarchical cluster analysis of all samples performed based on gene expression divided the twelve samples into three groups. The samples collected at 24 hpi (24_hpi_1, 24_hpi_2, and 24_hpi_3) and their corresponding controls (24_CK_1, 24_CK_2, and 24_CK_3) were grouped into separate clusters, while the samples collected at 48 hpi and their corresponding control samples were similarly grouped together (Fig. 3B). These results confirmed that the gene expression pattern of the resistant variety (QH1) was significantly altered at the early *Uv* infection stage (24 hpi). In the differential expressed gene (DEG) analysis, 1992 DEGs with 1060 upregulated genes were identified at 24 hpi (|logFC| > 1, adjusted p < 0.05; Fig. 3 C, Additional file 2: Table S2), while only 557 DEGs were found at 48 hpi, with 307 upregulated genes (Fig. 3 C, Additional file 3: Table S3).

### Functional clustering of all detected genes responsive to *Uv* infection

**Fig. 4:**
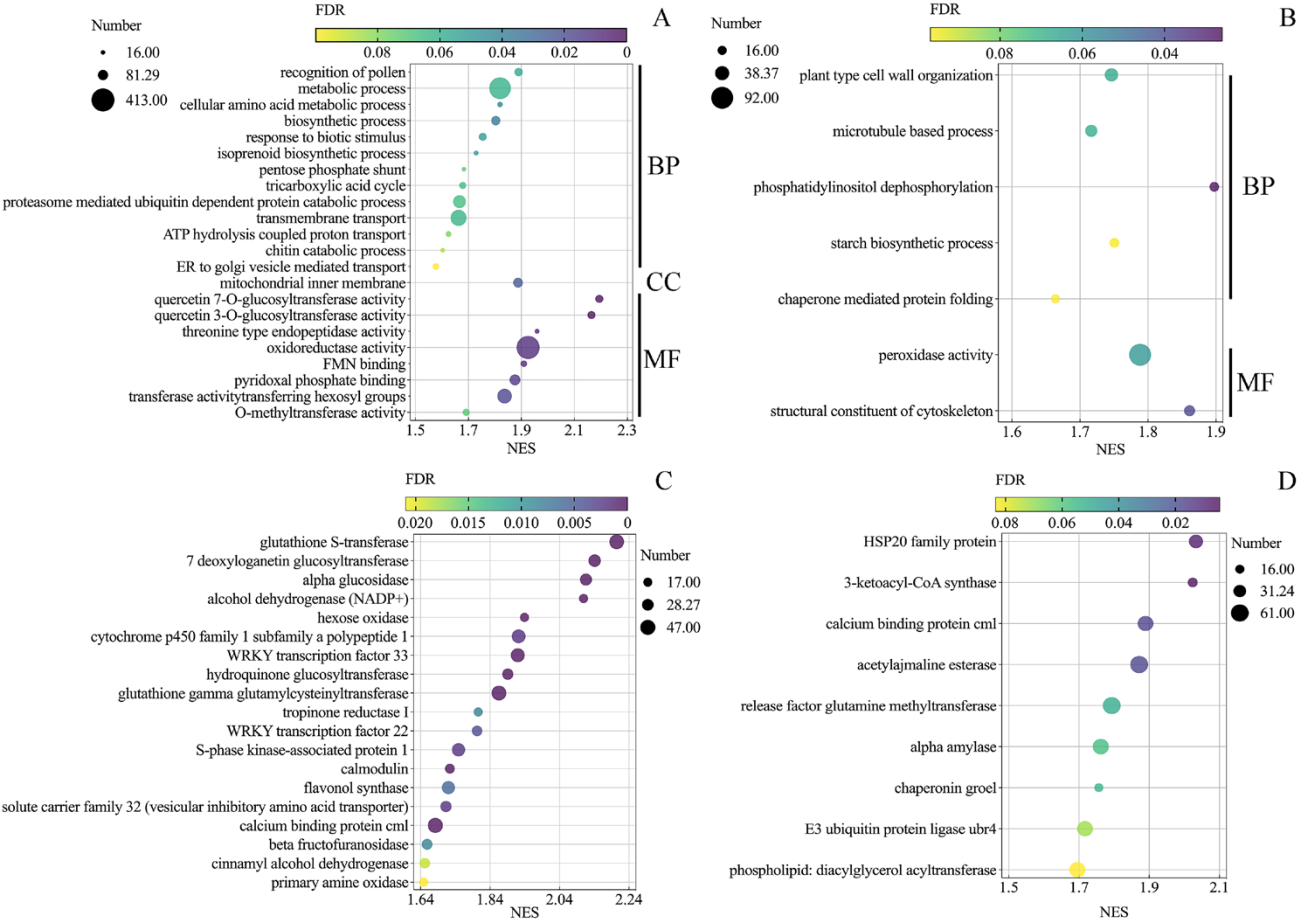
Gene ontology (GO) and KEGG pathway enrichment analysis of all detected genes at 24 and 48 hpi. (A-B), GO enrichment analysis results of all detected genes at 24 and 48 hpi. (C-D), KEGG enrichment analysis results of all detected genes at 24 and 48 hpi. GSEA: gene set enrichment analysis; NES: normalized enrichment score; Number: represented number of genes belonging to the related GO terms or pathways positively related to *Uv* infection at 24 and 48 hpi; FDR: false discovery rate (q-value). The GO terms or pathways with FDR values < 10% were considered to be enriched

To identify gene sets with statistically significant differences, gene ontology (GO) and Kyoto Encyclopedia of Genes and Genomes (KEGG) enrichment analyses were performed on all detected genes (n = 24,710) using gene set enrichment analysis (GSEA) software (false discovery rate (FDR) < 10%). Totals of 22 and 7 enriched GO terms were obtained at 24 (Fig. 4 A) and 48 hpi (Fig. 4B), respectively. At 24 hpi, the three most basic ‘biological process’ categories were the metabolic process, transmembrane transport, and proteasome-mediated ubiquitin-dependent protein catabolic process. Among the cellular components, only the mitochondrial inner membrane was enriched, and in the molecular function category, the three most-enriched categories were the oxidoreductase activity, transferase activity transferring hexosyl groups, and pyridoxal phosphate binding. In addition, the genes involved in biotic stress responses, including the chitin catabolic processes and responses to biotic stimuli, were also enriched. Among the 7 enriched GO terms identified at 48 hpi, most genes involved in cell wall organization and the structural constituents of cytoskeletons were enriched.

To investigate the biological pathways activated by *Uv* infection, the KEGG pathway enrichment was also analyzed (FDR < 0.1). Nineteen and 9 pathways were enriched at 24 (Fig. 4 C) and 48 hpi (Fig. 4D), respectively. Among the enriched pathways at 24 hpi, the top three pathways were glutathione S-transferase, the calcium-binding protein cml, and glutathione gamma glutamylcysteinyltransferase. At 48 hpi, the acetyladenine esterase pathway, release factor glutamine methyltransferase, and tRNA guanosine 2’-O-methyltransferase contained most of the enriched genes. Apart from the pathways with the greatest number of genes at different *Uv* infection stages, three pathways were commonly enriched, including the calcium-binding protein cml, hydroquinone glucosyltransferase, and WRKY transcription factor 22.

### qRT-PCR confirmed the RNA-seq results

To validate gene expression data obtained through differential expression analysis, relative expression of 10 DEGs were analyzed in the samples collected from QH1 at 24 and 48 hpi by qRT-PCR. Out of the 10 selected genes, 9 showed similar trends of expression, while the remaining one showed different trends (Additional file 4: Table S4). The results suggested that the gene expression information derived from the transcriptome is credible.

### DEGs associated with germination and invasion inhibition at the early stage of *Uv* infection

**Fig. 5:**
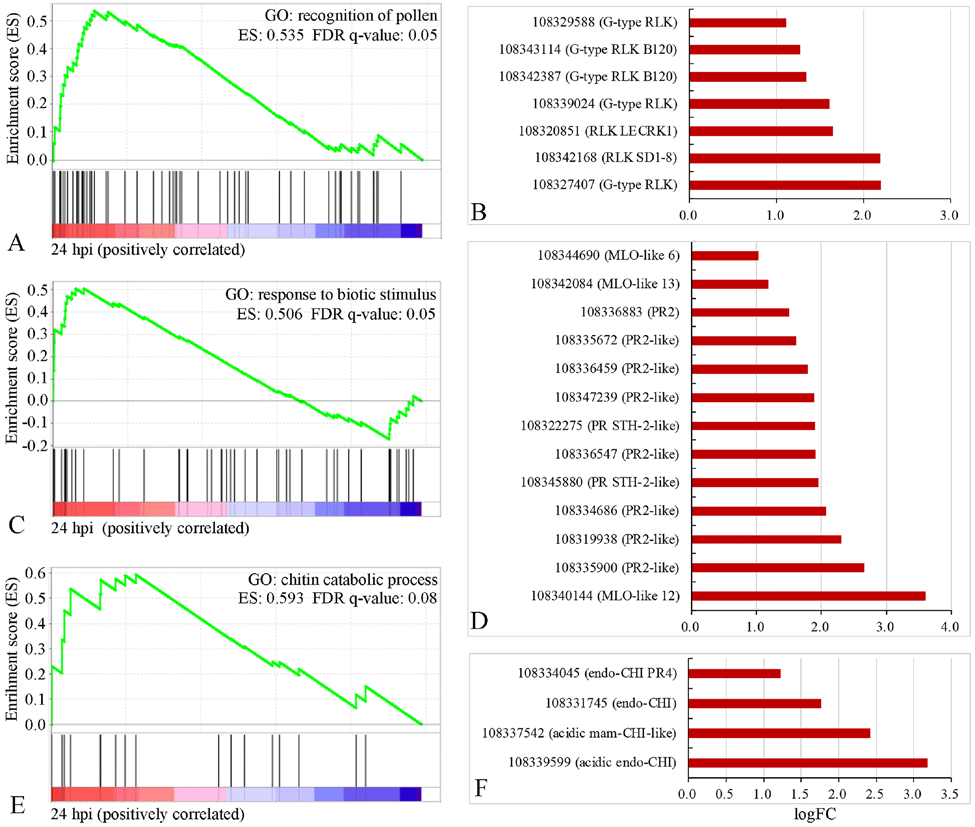
GSEA plot showing the most-enriched gene sets belonging to the three GO terms and their corresponding differentially expressed genes at 24 hpi. (A-B), recognition of pollen. (C-D), responses to biotic stimuli. (E-F), chitin catabolic process. G-type RLK: G-type lectin S-receptor-like serine/threonine-protein kinases; PR2: pathogenesis-related protein 2; Endo-CHI: endo-chitinase; Acidic mam-CHI: acidic mammalian chitinase; ES: enrichment score; FDR q-value: corrected p-value. An FDR value < 10% was considered to represent gene enrichment

Based on the fluorescence microscopy investigation, the obvious inhibition of uredospore development was investigated. Moreover, according to the enrichment analysis of the genes detected in the GSEA, we found that 19, 13, and 9 genes involved in the recognition of pollen, response to biotic stimulus and chitin catabolic process, respectively, were positively correlated with fungal resistance at 24 hpi (Fig. 5). Combined with the list of DEGs at 24 hpi, 7 G-type lectin S-receptor-like serine/threonine-protein kinases (G-type RLKs) out of the 19 genes correlated with pollen recognition were significantly upregulated (Fig. 5 A and B). In addition, 13 genes associated with biotic stimulus responses were significantly activated, including three Mildew Locus O (MLO)-like proteins and nine pathogenesis-related (PR) protein 2s (Fig. 5 C and D). Four genes encoding putative acidic endochitinase, acidic mammalian chitinase-like, and endochitinase were also induced (Fig. 5E and F).

According to the KEGG enrichment analysis performed using GSEA, we found two enriched pathways associated with WRKY transcription factors (TFs) 33 and 22 at 24 hpi. Combined with the list of DEGs at 24 hpi, six WRKY TFs were significantly induced by the *Uv* infections (Additional file 5: Table S5). Downstream of the WRKY TFs, PR proteins, including three PR1s, two PR2s, one PR4, two PR5s, twelve PR9s, two PR10s, and two PR14s (Additional file 5: Table S5), were significantly upregulated at 24 hpi.

### DEGs involved in cell wall modification and antimicrobial compound metabolites

**Fig. 6:**
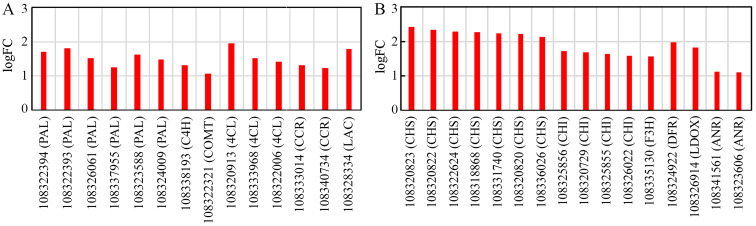
Genes involved in the lignin (A) and flavonoid (B) biosynthesis pathways were activated during *Uv* infection at 24 hpi. The upregulated genes of the corresponding pathways in infected leaves versus the control leaves at 24 hpi are listed. A, Upregulated genes involved in the lignin biosynthesis pathway. PAL: phenylalanine ammonia-lyase; C4H: cinnamate 4 hydroxylase; COMT: caffeic acid *O*-methyltransferase; 4CL: 4-coumarate-CoA ligase; CCR: cinnamoyl-CoA reductase; LAC: laccase. B, Upregulated genes involved in the flavonoid pathway. CHS: chalcone synthase; CHI: chalcone isomerase; F3H: flavanone 3-hydroxylase; DFR: dihydroflavonol 4-reductase; LDOX: leucoanthocyanidin dioxygenase; ANR: anthocyanidin reductase

Through fluorescence microscopy techniques, cell wall deposition and autofluorescence metabolite accumulation were observed during *Uv* spreading at 48 hpi (Fig. 2D). To explore the molecular bases of these processes, the DEGs associated with cell wall fortification and antimicrobial compound accumulation were identified. Among these DEGs, we found that several key genes contributing to phenolic compound biosynthesis were significantly induced at 24 hpi, including six phenylalanine ammonia-lyases (PALs), one cinnamate-4-hydroxylase (C4H), one caffeic acid O-methyl transferase (COMT), three 4-coumarate-CoA ligases (4CLs), two cinnamoyl-CoA reductases (CCRs), and a laccase (LAC) (Fig. 6 A). The upregulation of these enzyme-encoding genes may lead to the need for lignin biosynthesis and accumulation to cope with *Uv* spreading. Additionally, the genes that had been predicted to contribute to flavonoid production, including chalcone synthase (CHS), chalcone isomerase (CHI), flavanone 3-hydroxylase (F3H), dihydroflavonol 4-reductase (DFR), leucoanthocyanidin dioxygenase (LDOX), anthocyanidin reductase (ANR), and UDP-glucose flavonoid-3-O-glycosyltransferases (UFGTs), were also significantly upregulated (Fig. 6B), indicating dynamic flavonoid biosynthesis activity in response to *Uv* infection at 24 hpi.

In the later stage of *Uv* infection at 48 hpi, one CCR- and two peroxidase (POD)-encoding genes associated with lignin biosynthesis were found to be significantly upregulated (Additional file 6: Table S6). In addition, the gene encoding 3-ketoacyl-CoA synthase 1, which is involved in suberin biosynthesis, was also induced at 48 hpi (Additional file 6: Table S6). Apart from the genes contributing to cell wall fortification, a dihydroflavonol 4-reductase (DFR)-encoding gene was also activated at 48 hpi (Additional file 6: Table S6). At the same time, transcripts of eleven heat shock proteins (HSPs) were significantly accumulated at 48 hpi (Additional file 7: Table S7). According to the annotation and classification of HSPs in plants (reviewed in a previous study [20]), we found that HSP20 family proteins exhibited an increased responsibility for *Uv* spreading at 48 hpi. The expression levels of the 17.5-kDa class-I and 17.9-kDa class-II HSPs were most significantly increased and were upregulated 5.85- and 4.44-fold, respectively.

### qRT-PCR validation of the relative expression of defense-related genes

**Fig. 7:**
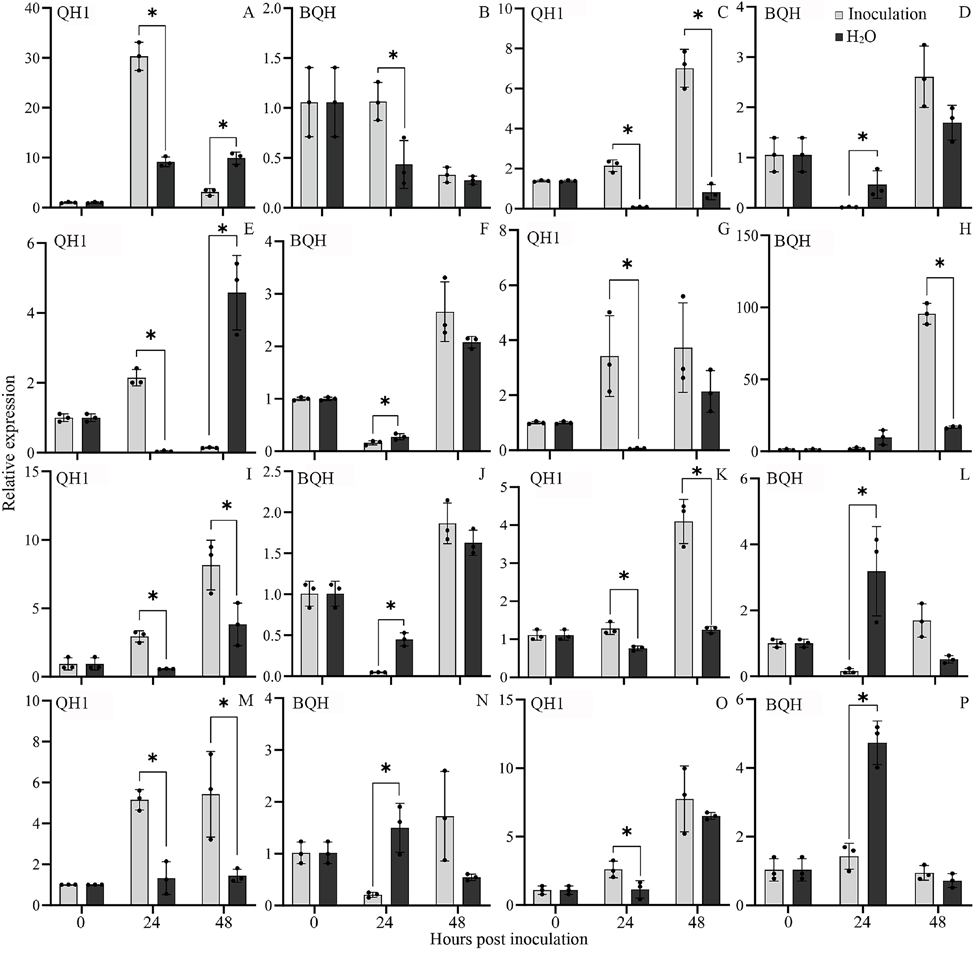
Expression of genes associated with chitinase and lignin biosynthesis by qRT-PCR. Samples collected both from QH1 and BQH at 24 and 48 hpi were used. (A-B), Relative expression of *PR4* (108,345,230). (C-D), Relative expression of *PAL* (phenylalanine ammonia-lyase, 108,326,061). (E-F), Relative expression of *C4H* (cinnamate 4 hydroxylase, 108,338,193). (G-H), Relative expression of *4CL* (4-coumarate-CoA ligase, 108,322,006). (I-J), Relative expression of *CCR* (cinnamoyl-CoA reductase, 108,333,014). (K-L), Relative expression of *LAC* (laccase, 108,328,334). (M-N), Relative expression of the gene encoded peroxidase P7-like (108,322,789). (O-P), Relative expression of the gene encoded peroxidase 5-like (108,322,789). * Represents significantly difference between the infected (Inoculation) and uninfected (H_2_O) samples (*P* < 0.05)

To confirm the potential role of the genes in rust resistance, expression analysis of genes associated with chitinase activity, and lignin biosynthesis in both QH1 and BQH at 24 and 48 hpi was conducted by qRT-PCR. For *PR4*, related to the chitinase activity, the relative expression was significantly induced by *Uv* infection both in QH1 and BQH (Fig. 7 A and B) at 24 hpi. But the relative expression level of *PR4* in QH1 was much higher than that in BQH. In addition, transcripts of the genes involved in lignin biosynthesis such as *PAL*, *C4H*, *4CL*, *CCR*, *LAC*, and *POD* (peroxidase P7-like, and peroxidase 5-like) were also significantly accumulated in QH1 by *Uv* infection (Fig. 7 C to P). In addition, in resistant cv. QH1, either the expression level of these genes was higher, or the responsive time was earlier than that in BQH.

**Fig. 8:**
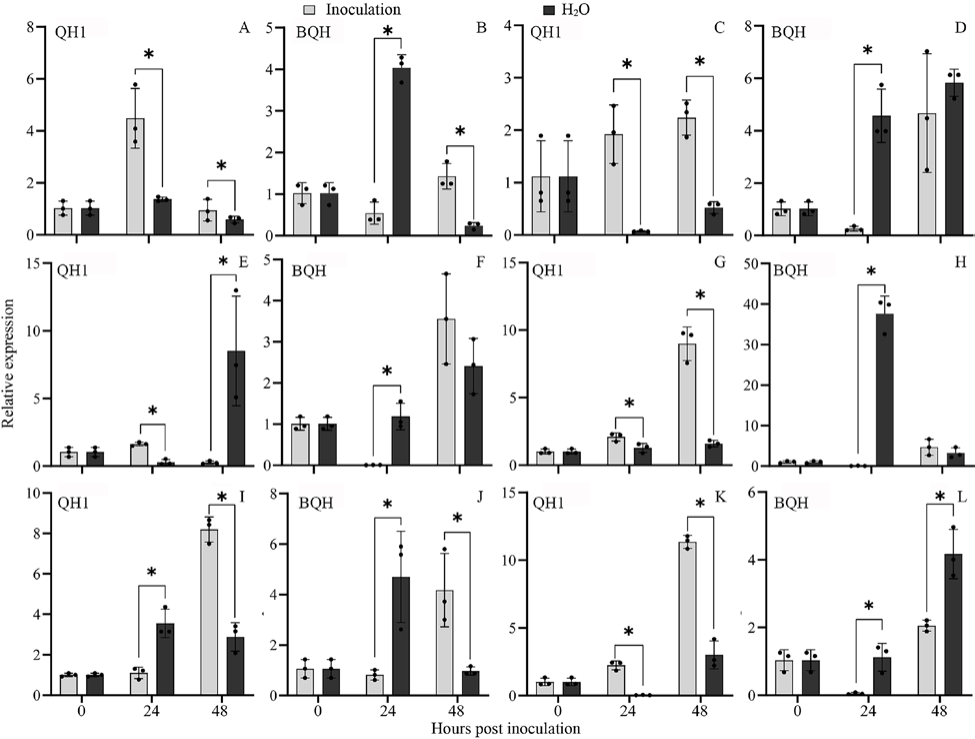
Expression of genes involved in flavonoid biosynthesis by qRT-PCR. Samples collected both from QH1 and BQH at 24 and 48 hpi were used. (A-B), Relative expression of *CHS* (chalcone synthase, 108,331,740). (C-D), Relative expression of *CHI* (chalcone isomerase, 108,325,855). (E-F), Relative expression of *F3H* (flavanone 3-hydroxylase, 108,335,130). (G-H), Relative expression of *DFR* (dihydroflavonol 4-reductase, 108,324,448). (I-J), Relative expression of *LDOX* (leucoanthocyanidin dioxygenase, 108,326,914). (K-L), Relative expression of *ANR* (anthocyanidin reductase, 108,341,561). * Represents significantly difference between the infected (Inoculation) and uninfected (H_2_O) samples (*P* < 0.05)

DEGs such as *CHS* (108,331,740), *CHI* (108,325,855), *F3H* (108,335,130), *DFR* (108,324,448), *LDOX* (108,326,914), and *ANR* (108,341,561) involved in flavonoid biosynthesis were also selected for expression analysis in QH1 and BQH through qRT-PCR (Fig. 8). The results showed that *Uv* infection increased of transcripts for these genes in resistant cv. QH1 rather than the susceptible cv. BQH. Furthermore, genes such as *CHS*, *CHI*, *DFR*, and *ANR* were highly upregulated in resistant cv. QH1 at 24 and 48 hpi but suppressed in susceptible cv. BQH. These findings support the reliability of transcriptome-based gene expression data while also raising the possibility that these genes have a significant impact on rust resistance.

### Chitinase and phenylalanine ammonia-lyase activity are significantly increased in the resistant variety

RNA sequencing and qRT-PCR analysis showed that genes encoding chitinase (CHI) and phenylalanine ammonia-lyase (PAL) were significantly induced in *Uv-*infected cv. QH1. To confirm the roles of the corresponding enzymes in *Uv* resistance, the activities of CHI and PAL in different resistant varieties infected with *Uv* were investigated.

**Fig. 9:**
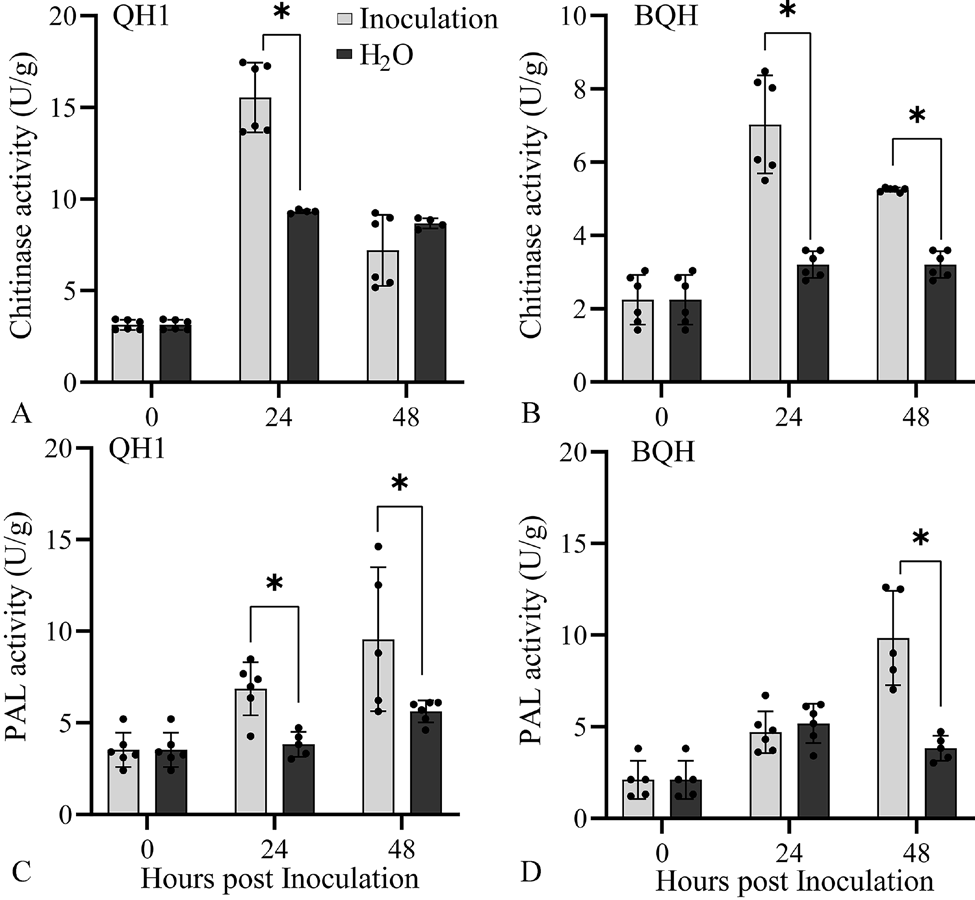
Chitinase (CHI) and phenylalanine ammonia-lyase (PAL) activity in the resistant (QH1) and susceptible (BQH) cultivars at 24 and 48 hpi. A and B, CHI activities in leaves of QH1 (A) and BQH (B) inoculated with *Uv* (Inoculation) and sterile water (H_2_O). C and D, PAL activities in leaves of QH1 (C) and BQH (D) inoculated with *Uv* (Inoculation) and sterile water (H_2_O). Data represent means ± S.D. of six replicate samples. *, significant difference between inoculation and control at *P* < 0.05 based on two independent sample T test

Compared with the uninfected control, CHI activity was dramatically elevated at 24 hpi in resistant cv. QH1 (Fig. 9 A), which corresponded to the transcript accumulation of CHI-encoding genes (Fig. 5E and F). Although CHI activity was also induced by *Uv* at 24 and 48 hpi in susceptible cv. BQH (Fig. 9B), CHI activity was 2.21 times and 1.37 times higher, respectively, in the resistant cv. QH1 than in the susceptible cv. BQH.

Compared with the uninfected control, PAL activity in the leaves of resistant cv. QH1 was significantly induced by *Uv* infection at 24 and 48 hpi (Fig. 9 C). These results confirmed the upregulation of transcripts for PAL-encoding genes detected by transcriptome sequencing (Fig. 6 A). Furthermore, to determine the role of PAL in *Uv* resistance, we compared the changes in PAL activity in susceptible cv. BQH inoculated with *Uv*. The results showed that PAL activity was also significantly increased at 48 hpi (Fig. 9D). However, the average increase compared with the control occurred much earlier in resistant cv. QH1 (24 hpi) than in susceptible cv. BQH (48 hpi). In addition, PAL activity was 1.46 times higher in resistant cv. QH1 than in susceptible cv. BQH.

## Discussion

Adzuki bean rust is caused by *U. vignae* and is the most devastating disease affecting adzuki bean production in China. To better understand this pathosystem, we investigated the fungal infection characteristics in differently resistant cultivars. In addition, time-resolved RNA sequencing of the leaves of resistant cultivar seedlings were also conducted to analyze the transcriptional changes associated with *Uv* infection.

The pathogenic infection characteristics of *Uv* on adzuki bean leaves of different resistant cultivars were compared at two time points (24 and 48 hpi) corresponding to the timing of the fungal entry through the stomata and the asymptomatic colonization of mesophyll tissue by intercellular haustoria formation (Fig. 2). The uredospore-based inoculation of *Uv* onto the leaves led to symptoms that varied significantly in severity on different cultivars, as shown in Fig. 1. Further fluorescence microscopy investigations showed that the uredospore germination frequency was significantly lower on QH1 than that on BQH. Additionally, although the germinated fungus entered the substomatal cavity by developing Sv at 24 hpi, most of these Sv malformed and failed to generate Ih (Fig. 2 B). During the interaction between wheat and *U. fabae* (*Uf*), the *Uf* on the wheat leaf surface can form appressoria over stomata, but germ tubes rarely enter the substomatal cavity or develop haustoria [21]. In addition, significant levels of aborted stomatal penetration and reduction of haustoria formation were found on all resistant lines of faba bean in response to *U. viciae-fabae* infection [22]. Similarly, lower uredospore germination, and higher abortion of infection structures were observed in an incompatible interaction between *Medicago truncatula* and *U. viciae-fabae*, and *U. lupinicolus* [23]. These results indicated that the inhibition of *Uv* uredospore germination, invasion, and spreading were the important reasons for the high resistance of QH1.

To gain insight into the molecular basis of QH1 resistance to *Uv* infection, a high-sensitivity RNA-seq gene expression profiling approach was utilized. According to the enrichment characteristics and DEG analysis results, we found that the number of genes encoding G-type RLKs was significantly induced by *Uv* invasion at 24 hpi in the resistant cv. QH1. The G-type RLKs known as S-domain RLKs are characterized by extracellular lectin motifs [24]. Due to the resemblance of this extracellular domain to lectin proteins known to bind to fungal and bacterial cell wall components, G-type RLKs are predominantly hypothesized to be involved in biotic stress [25]. For example, the G-type RLK *NbLRK1* in *Nicotiana benthamiana* is a component of the *N. benthamiana* protein complex that recognizes the *Phytophthora infestans* INF1 elicitor and subsequently mediates INF1-induced cell death [26]. *Pi-d2*, a G-type RLK from rice, provides resistance against the fungal pathogen *Magnaporthe grisea* [27]. These results imply that G-type RLKs in resistant cv. QH1 serve as cell surface pathogen recognition receptors (PRRs).

PRRs are considered to play important roles in initiating plant innate immune responses by directly recognizing pathogen-associated molecular patterns (PAMPs) [28]. As key regulators in the immune responses of plants to a variety of biotic stresses, WRKY TFs have been reported to play pivotal roles in PAMPs-triggered immunity (PTI) through direct or indirect interactions with PAMPs proteins or their regulatory proteins [29]. *OsWRKY62* has been demonstrated to participate in PTI defense against *Xanthomonas oryzae* pv. *oryzae* (Xoo) in rice [30]. In addition, *OsWRKY67* has been indicated to act as a positive PTI regulator in rice against two rice pathogens, *M. oryzae* and Xoo [31]. In the current study, we found six WRKY TFs in the resistant cv. QH1 that were activated by *Uv* infection at 24 hpi, suggesting that the WRKY TFs in cv. QH1 might play positive roles in the PTI response triggered by G-type RLKs. As key regulators in the plant immune response, WRKY TFs regulate the expression of defense-related genes by binding to a consensus cis-element referred to as the “W-box” in the promoter regions of these genes [32, 33]. The latest discoveries illustrate that WRKY interacts with other TFs to form integral signaling network components that regulate the defense-related genes that contribute to the immune response of plants [29]. In this study, the number of *PRs* was also found to be significantly induced by *Uv* infection at 24 hpi, including *PR1*, *PR2*, *PR4*, *PR5*, *PR9*, *PR10*, and *PR14* (Fig. 5 and Additional file 5: Table S5). *PR2* encodes (1,3)-β-glucanase, which has an inhibitory effect against a wide range of fungi [34, 35] and usually acts in synergy with CHI [35]. In addition, the PR1 and PR5 proteins are considered the best source of antimicrobial substances to help plants evade pathogenic infections [35, 36]. A previous study revealed that PR5 is involved in acquired systemic resistance and response to biotic stress, thus inhibiting hyphal growth and reducing spore germination, likely through a membrane-permeabilization mechanism [37]. Moreover, twelve PR9 proteins that encode peroxidase, which is involved in lignin and suberin formation, the cross-linking of cell wall components, the synthesis of phytoalexin, and the biosynthesis of reactive oxygen species (ROS), were significantly upregulated at 24 hpi (Additional file 5: Table S5) [35]. Accordingly, the accumulation of these antifungal proteins and phytoalexin in the resistant cv. QH1 at 24 hpi might have been responsible for the significant decrease in uredospore germination and the increase in Sv deformities (Fig. 1 C). Moreover, the induced expression of several peroxidase-encoding genes indicated that the formation of ROS may potentiate further activation of defensive genes.

Another prominent response observed during *Uv* infection in resistant cv. QH1 was the strong activation of lignin biosynthesis genes; phenylalanine ammonia-lyase, cinnamate-4-hydroxylase, caffeic acid *O*-methyltransferase, 4-coumarate-CoA ligase, cinnamoyl-CoA reductase, peroxidase, and laccase genes were highly expressed (Fig. 6 A). In parallel, the increased expression of the enzymes important for reinforcing cell walls by lignification began at 24 hpi and further increased at 48 hpi (Additional file 6: Table S6). Phenylpropanoid-derived lignin acts by cross-linking plant secondary cell walls to provide mechanical defensive strength [38]. These results were similar to those of a previous study on the interaction between barley and *Ramularia collo-cygni*; in that study, genes such as caffeic acid *O*-methyltransferase and 4-coumarate-CoA ligase were also found to be significantly highly expressed [39]. In addition to lignin formation, phenolic compounds used for flavonoid biosynthesis were also utilized by adzuki beans to cope with *Uv* infection. Flavonoids are usually considered to act as phytoalexins with antifungal properties and are produced in response to pathogen attacks [40]. A strong increase in the expression of adzuki bean genes encoding key flavonoid pathway enzymes was identified at 24 and 48 hpi in the *Uv*-inoculated leaves (Fig. 6B, Additional file 6: Table S6). The results demonstrated that the resistant cv. QH1 likely reflected an attempt to control *Uv* colonization by producing flavonoid phytoalexins. Congruent with the gene expression characteristics, the *Uv* invasion and intracellular spread were significantly inhibited by cell wall fortification and antimicrobial phenolic compound accumulation (Fig. 2B and D). Our RNA-seq analysis also revealed that the numbers of genes encoding small HSPs were highly modulated at 48 hpi. Small HSPs are a large and ancient family of proteins that are regulated in response to biotic stress and are among the adaptive systems in plants that interact with phytopathogenic organisms [41]. The knockdown of some small HSPs in rice has been shown to affect the severity of *Magnaporthe grisea* infections in some cases [42]. When the tomato HSP20 gene was silenced, a more severe *Fusarium oxysporum* infection was noted [43]. In Arabidopsis, HSPs, along with PR proteins, have been shown to be most effective against *Rhizoctonia solani*. The HSP20 expression, along with the PR protein expression, was more than 10-fold in the pathogen-resistant genotype AG-8 but was normal in the susceptible genotype AG2-1 in a previous study [44, 45]. Based on the upregulation of HSPs and PRs during *Uv* infections, it is likely that HSPs, along with other defense-related genes, contribute to the resistance of adzuki beans against rust fungal infection.

Genes encoding CHI and PAL were significantly enriched and upregulated in QH1 according to RNA sequencing, which confirmed the correlation between gene expression and corresponding enzyme activities in *Uv* resistance. CHI and PAL activities in resistant cv. QH1 and susceptible cv. BQH were compared (Fig. 7). The results showed that CHI and PAL activities in leaves were generally higher in resistant cv. QH1 than in susceptible cv. BQH, and the average increase compared with the control occurred earlier in QH1 than in BQH. Based on these changes in gene expression levels and enzyme activity, we speculated that high level and timely response of defense-related genes in the process of *Uv* infection may be an important reason for the resistance of QH1.

## Conclusion

In conclusion, cell surface PRRs in adzuki beans exposed to *Uv* infections initiate defensive responses and subsequently activate antimicrobial PRs by regulating the transcript levels of WRKY TFs. We believe that this is a critical reason why the uredospore germination rate and infection rate obviously decreased on the leaves of the resistant cv. QH1. Resistance reaction in QH1 to *Uv* infection including inhibition of uredospore germination, Sv development, cell wall deposition and autofluorescence around the infection sites. Congruent with these defense responses, the gene transcripts associated with lignin, suberin, and antimicrobial phenolic compound biosynthesis were significantly increased, and defense-related enzyme were significantly activated, including CHI and PAL. These analyses, which combined both histological and molecular responses, provide increased insight into the molecular resistance mechanisms of adzuki bean when facing *Uv* infections. Collectively, our study provides important basis for further exploring the molecular mechanism of rust resistance of adzuki bean. It helps to identify the potential key rust resistance genes for the genetic manipulation of resistant adzuki bean cultivars in the future.

## Methods

### Adzuki bean varieties and *Uv* isolate

The two varieties of adzuki bean, the QH1 (resistant) and Baoqinghong (BQH, susceptible), were obtained from National Coarse Cereals Engineering Research Center, Heilongjiang Bayi Agricultural University. The *Uromyces vignae* isolate ZXL01 [7] used in this study was obtained from the Plant Immunity Laboratory of the College of Agriculture, Heilongjiang Bayi Agricultural University.

### Adzuki bean seedling cultivation, inoculation, and leaf sample collection

The seedlings of both the varieties were raised in growth chambers under controlled conditions with 16 h light (200 µmol m^− 2^ s^− 1^) at 23 ± 2 °C and 8 h of darkness at 19 ± 2 °C at Heilongjiang Bayi Agricultural University. In the inoculation process, fresh *Uv* uredospore suspensions (1 × 10^5^ uredospore mL^− 1^) were sprayed on the unifoliolate leaves of 8-day-old seedlings using a hand sprayer according to a previously described protocol [7]. Parallel mock inoculations were performed with sterile water. The inoculated seedlings were kept in a humid chamber for 24 h at 20 ± 2 °C in complete darkness and then moved to growth chambers. Leaf samples were collected at specific time points for various analyses. The remaining seedlings were grown to confirm the symptoms of fungal infection. The resistance reactions of both cultivars were evaluated by observing the initial time of symptom occurred, sporulation and the number of final uredia formed on the leaf surface.

### Histological analysis of *Uv* infection in different resistant cultivars

Three inoculated leaves were collected from the BQH and QH1 cultivars at 24 and 48 hpi for the histopathological analysis. Adzuki bean leaf pieces with sizes of 2–3 cm^2^ were cut from the centers of the inoculated leaves. The leaf sections were processed following the methods described by Zhang et al. [46]. The leaf segments were fixed and decolorized in ethanol/trichloromethane (3:1 v/v) containing 0.15% (w/v) trichloroacetic acid for 3–5 days. The specimens were then cleaned in saturated chloral hydrate until the leaf tissues became translucent. For the Calcofluor staining, the cleared leaf segments were washed twice with 50% (v/v) ethanol for 15 min. The leaves were then rinsed with water and soaked in 0.5 M NaOH. After washing with water, the specimens were incubated in 0.1 M Tris-HCl (pH 8.5) for 30 min and then stained with 0.1% (w/v) Calcofluor white M2R (product number 910,090, Sigma–Aldrich) for 15 min. After being washed with water four times (for 10 min each) and once (for 30 min) with 25% (v/v) glycerol, the specimens were examined with an Olympus BX-60 microscope (Olympus Corporation). For the leaf samples harvested at 24 hpi, approximately 100–120 uredospores from 6 to 8 leaf segments were examined to determine the germination frequency, and 30 infection sites from 6 to 8 leaf segments were examined to determine the infection frequency and substomatal vesicle (Sv) deformity rate. The formation site of a Sv was defined as an infection site.

### Transcriptomic analysis of resistant cv. QH1 response to *Uv* infection

For the transcriptomic analysis, leaf samples were collected from the QH1 cultivar at 24 and 48 hpi in triplicate and used for RNA sequencing. The total RNA of the samples was derived using the RNeasy Micro kit (Qiagen, Shenzhen, PRC) according to the manufacturer’s protocol. The RNA sequence libraries were prepared using the NEBNext® Ultra™ RNA Library Prep Kit for Illumina® (New England Biolabs, Inc.) and sequenced on an Illumina HiSeq2500 platform (50 bases, single end) designed by Beijing Novogene Technology Co., Ltd. (Beijing, China). The raw data were trimmed of adaptors and low-quality reads using an in-house (Novogene) Perl script. The raw data were then deposited in the National Center for Biotechnology Information Sequence Read Archive database under BioProject PRJNA431103. All downstream analyses were based on these high-quality clean data.

The RNA-seq data were mapped to the published adzuki bean genome [47] using hierarchical indexing for spliced alignment of transcripts (HISAT) v2.0.4 [48]. In the gene expression profiling process, the fragments per kilobase of transcript per million fragments mapped (FPKM) value of each gene was calculated using RNA-seq by expectation-maximization (RSEM) v1.2.12 [49]. Differential gene expression was calculated using the R package DESeq v1.10.1 [50] while considering the independent biological sample triplicates. In our downstream analyses, we included adzuki bean genes that were differentially expressed (DEGs) (|log2(FoldChange)| > 1 and a Q-value < 0.005) compared with the uninoculated leaves at the corresponding time points.

Gene ontology (GO) and Kyoto Encyclopedia of Genes and Genomes (KEGG) pathway [51] enrichment analyses of all detected genes were conducted using Gene Set Enrichment Analysis (GSEA) software version 4.1.0 [52]. GO terms and KEGG pathways with FDR q-values (corrected *P value*s) less than 10% were considered significantly enriched.

### Quantitative RT-PCR analysis

For qRT-PCR, leaf samples were collected from QH1 and BQH at 24 and 48 hpi in triplicate and used for gene expression analysis. Total RNA of the collected samples from QH1 and BQH were isolated by the RNeasy Micro kit (Qiagen, Shenzhen, PRC) according to the manufacturer’s protocol. And the cDNA synthesized by the FastQuant RT Kit with gDNase (KR106, TianGen, Beijing, China). To confirm expression data of RNA sequencing, samples of QH1 were used for a set of 10 selected DEGs expression analysis. In addition, both QH1 and BQH samples were used for expression validation of the genes associated with rust resistance. Actin encoding gene *VaACT* was used as internal reference [53]. PCR was performed in a 10 µL reaction mixture with 5 µL SYBR Premix Ex Taq (Takara, Japan), 0.2 µL of both forward and reverse primers, 3.6 µL of double-distilled H_2_O and 1 µL (40 ng/µL) of the cDNA. qRT-PCR was performed using SYBR Green in a qTOWER 3G system (Jenna). The thermal cycler conditions were 95° for 5 min, followed by 40 cycles of 95° for 10 s, 60° for 20 s, and 72° for 20 s. All qRT-PCR experiments were performed in triplicate using independent samples. Relative expression was calculated according to the 2^−ΔΔCt^ method [54]. Primers used in current study were designed by Primer3 [55] and listed in Additional file 8: Table S8.

### Chitinase and phenylalanine ammonia-lyase activity in different resistant cultivars response to *Uv* infection

According to the transcriptomic data obtained from the resistant cv. QH1 in response to Uv infection, we found that genes encoding chitinase (CHI) and phenylalanine ammonia-lyase (PAL) were significantly induced by *Uv* infection. To confirm the roles of CHI and PAL enzymes in adzuki bean resistance to *Uv* infection, leaf samples of cv. QH1 and cv. BQH inoculated with *Uv* were collected at 0, 24, and 48 hpi for determination of enzyme activity.

PAL activity was assayed spectrophotometrically following the method of Kosuge and Conn [56] with slight modifications, and 6 biological replicates were conducted. Leaf samples (0.1 g) from different treatments were homogenized in a chilled mortar with 5% polyvinylpyrrolidone (PVP) and 2 mL of 100 mM Tris–HCl buffer (pH 7.5) containing 14 mM β-mercaptoethanol, 5 mM DL-dithiothreitol, 10% Glycerol, 0.5% Triton X-100, and 1% bovine serum albumin (BSA). Afterward, 1 mL of supernatant was applied to a PD10 column equilibrated with 100 mM Tris–HCl (pH 7.5) buffer. Protein was eluted with 2 mL of elution buffer. The enzyme extract was used for immediate analysis. Activity was determined spectrophotometrically by measuring the amount of trans-cinnamic acid formed at 290 nm (extinction coefficient of 17.4 mM^− 1^ cm^− 1^). A reaction mixture that lacked L-phenylalanine was used as control.

For CHI, with 6 biological replicates, leaf samples (0.1 g) were homogenized with a chilled mortar and pestle in 2 mL of 100 mM sodium citrate buffer (pH 5.0) containing 1 mM EDTA and 5 mM β-mercaptoethanol. The homogenate was centrifuged for 10 min at 10,000 g. Afterward, 1 mL of the supernatant was desalted on a PD10 column (GE Healthcare, Buckingharmshire, UK) using 100 mM sodium citrate buffer (pH 5.0) for elution. We collected the first 2 mL after the void volume for the chitinase assay. The chitinase activity assay was performed in strict accordance with the instructions of the Chitinase Activity Detection Kit (BC0820, Beijing Solarbio Science & Technology Co., Ltd, China). Activity was determined spectrophotometrically by measuring the absorbance at 540 nm in an ultraviolet-visible light spectrophotometer (HITACHI U-2910).

## Electronic supplementary material

Below is the link to the electronic supplementary material.

Supplementary Material 1

Supplementary Material 2

Supplementary Material 3

Supplementary Material 4

Supplementary Material 5

Supplementary Material 6

Supplementary Material 7

Supplementary Material 8

## Data Availability

The RNA-seq data utilized in this article are available from the National Center for Biotechnology Information (NCBI) Sequence Read Archive Database at [https://www.ncbi.nlm.nih.gov/bioproject/PRJNA431103] and can be accessed with [BioProject PRJNA431103].

## Abbreviations

*Uv*: Uromyces vignae
DEGs: differentially expressed genes
hpi: hours post-inoculation
PAMPs: pathogen-associated molecular patterns
PRRs: pattern recognition receptors
PTI: PAMPs-triggered immunity
PR: pathogenesis-related
Um: suredium
Ih: infection hyphae
Hm: haustoria mother cell
Ha: haustoria
GO: gene ontology
BP: biological processes
CC: cellular component
MF: molecular function
RLPs: receptor-like proteins
RLK: receptor-like kinase
PAL: phenylalanine ammonia-lyase
C4H: cinnamate-4-hydroxylase
COMT: caffeic acid *O*-methyltransferase
4CL: 4-coumarate-CoA ligase
CCR: cinnamoyl-CoA reductase
LACs: laccases
CHS: chalcone synthase
CHI: chalcone isomerase
F3H: flavanone 3-hydroxylase
DFR: dihydroflavonol 4-reductase
LDOX: leucoanthocyanidin dioxygenase
ANR: anthocyanidin reductase
UFGTs: UDP-glucose flavonoid-3-*O*-glycosyltransferases
FPKM: Fragments Per Kilobase of transcript per Million fragments mapped
GSEA: gene set enrichment analysis
