# Supplementary material for: Histological and molecular responses of Vigna angularis to Uromyces vignae infection
Source: BMC Plant Biol. 2022 Oct 14;22:489. doi: 10.1186/s12870-022-03869-2 (PMC9563176; doi:10.1186/s12870-022-03869-2)
Supplement: Supplementary file 1 — Supplementary Material 1 [file 12870_2022_3869_MOESM1_ESM.docx]

**Table S1.** Basic summary of sequencing and clean reads mapping to the reference genome

| Sample | Total Clean Reads | Total Mapping Ratio | Uniquely Mapping Ratio |
| --- | --- | --- | --- |
| 24_CK_1 | 8151148 | 97.27% | 87.92% |
| 24_CK_2 | 7840315 | 97.14% | 88.07% |
| 24_CK_3 | 7591059 | 97.31% | 88.67% |
| 48_CK_1 | 10316722 | 97.31% | 88.20% |
| 48_CK_2 | 9817795 | 97.37% | 88.24% |
| 48_CK_3 | 8946516 | 97.98% | 88.93% |
| 24_hpi_1 | 10706005 | 97.10% | 87.95% |
| 24_hpi_2 | 9344742 | 97.28% | 87.95% |
| 24_hpi_3 | 8713338 | 97.19% | 87.87% |
| 48_hpi_1 | 9400103 | 97.16% | 87.90% |
| 48_hpi_2 | 10066943 | 97.43% | 88.25% |
| 48_hpi_3 | 9101189 | 97.79% | 88.43% |

Total Clean Reads: Filtered sequencing data of each sample; Total Mapping Ratio: Frequency of the reads that can be located on the *Vigan angularis* genome; Uniquely Mapping Ration: Frequency of the reads that has a unique alignment position on the reference sequence.
