# Supplementary material for: Histological and molecular responses of Vigna angularis to Uromyces vignae infection
Source: BMC Plant Biol. 2022 Oct 14;22:489. doi: 10.1186/s12870-022-03869-2 (PMC9563176; doi:10.1186/s12870-022-03869-2)
Supplement: Supplementary file 4 — Supplementary Material 4 [file 12870_2022_3869_MOESM4_ESM.docx]

**Table S4.** Expression validation of the DEGs by qRT-PCR

| Gene_ID | log2FC ^a^ | Log2ratio ^b^ | Concordant ^c^ |
| --- | --- | --- | --- |
| DEGs from 24 hpi | | | |
| 108322711 | 1.77 | 1.56±0.01 | Yes |
| 108337247 | 2.34 | 2.11±0.04 | Yes |
| 108322622 | 4.90 | 4.08±0.13 | Yes |
| 108324651 | 2.63 | -0.17±0.01 | No |
| DEGs from 48 hpi | | | |
| 108337322 | 5.85 | 70.16±0.14 | Yes |
| 108342175 | -2.94 | -1.96±0.05 | Yes |
| 108325713 | 1.70 | 1.85±0.02 | Yes |
| 108335120 | 2.04 | 2.59±0.23 | Yes |
| 108346982 | -1.22 | -1.56±0.11 | Yes |
| 108321079 | 1.02 | 3.01±0.03 | Yes |

**^a^** Gene expression data obtained from RNA-Seq, Log2FC represent the relative expression level of the DEGs from 24_hpi and 48_hpi, respectively. **^b^** Data of qRT-PCR obtained through statistical comparison of the genes expressed differentially at 24_hpi and 48_hpi, respectively. The data of Log2ratio was the mean value of relative transcript level of each gene in three replicates and calculated by log2 (expression level after inoculation with *U. vignae* / expression level after inoculation with sterile water). **^c^** ‘Yes’ indicates that gene expression verified by qPCR was consistent with differential expression analysis by RNA-Seq, while ‘No’ indicates the gene expression data did not correlate with each other.
