# Supplementary material for: Histological and molecular responses of Vigna angularis to Uromyces vignae infection
Source: BMC Plant Biol. 2022 Oct 14;22:489. doi: 10.1186/s12870-022-03869-2 (PMC9563176; doi:10.1186/s12870-022-03869-2)
Supplement: Supplementary file 5 — Supplementary Material 5 [file 12870_2022_3869_MOESM5_ESM.docx]

**Table S5.** Differentially expressed WRKY transcription factors and pathogenesis-related proteins at 24 hpi

| Gene_ID | logFC | q-value | Description |
| --- | --- | --- | --- |
| 108332713 | 4.96 | 2.13E-05 | Probable WRKY transcription factor 70 |
| 108341502 | 2.17 | 5.81E-227 | Probable WRKY transcription factor 26 |
| 108328346 | 2.12 | 0 | Probable WRKY transcription factor 75 |
| 108320588 | 1.42 | 0 | Probable WRKY transcription factor 40 |
| 108347033 | 1.32 | 3.56E-48 | Probable WRKY transcription factor 33 |
| 108329829 | 1.26 | 9.79E-08 | Probable WRKY transcription factor 61 |
| 108342448 | 2.73 | 0 | Pathogenesis-related protein 1-like |
| 108342391 | 2.41 | 0 | Pathogenesis-related protein 1-like |
| 108342629 | 4.71 | 5.28E-08 | Basic form of pathogenesis-related protein 1-like |
| 108333874 | 1.59 | 7.18E-177 | Endo-1,3;1,4-beta-D-glucanase-like (PR2) |
| 108336423 | 7.53 | 1.08E-23 | Pathogenesis-related protein 2-like |
| 108345230 | 1.82 | 0 | Pathogenesis-related protein PR-4-like |
| 108325249 | 1.55 | 0 | Thaumatin-like protein 1b (PR5) |
| 108335438 | 1.22 | 0 | Thaumatin-like protein 1 (PR5) |
| 108343028 | 3.18 | 0 | Peroxidase E5-like (PR9) |
| 108324140 | 2.40 | 0 | Peroxidase 12-like (PR9) |
| 108328319 | 1.58 | 0 | Cationic peroxidase 1-like (PR9) |
| 108327974 | 1.08 | 1.40E-179 | Cationic peroxidase 1-like (PR9) |
| 108343153 | 3.18 | 8.25E-114 | Cationic peroxidase 1-like (PR9) |
| 108327706 | 1.99 | 9.18E-101 | Peroxidase 55-like (PR9) |
| 108319204 | 1.38 | 2.76E-20 | Peroxidase 21 (PR9) |
| 108338053 | 3.24 | 5.51E-20 | Cationic peroxidase 1-like (PR9) |
| 108344790 | 1.08 | 6.13E-13 | Peroxidase 47-like (PR9) |
| 108339962 | 5.49 | 2.90E-07 | Lignin-forming anionic peroxidase-like (PR9) |
| 108340339 | 1.51 | 5.50E-05 | Lignin-forming anionic peroxidase-like (PR9) |
| 108335606 | 1.51 | 9.60E-05 | Peroxidase 5-like (PR9) |
| 108338105 | 3.52 | 5.63E-96 | Extracellular ribonuclease LE-like (PR10) |
| 108335416 | 1.81 | 0.00065 | Ribonuclease 3-like (PR10) |
| 108333619 | 1.40 | 0 | Nonspecific lipid-transfer protein P5 (PR14) |
| 108341563 | 1.01 | 1.42E-14 | Nonspecific lipid-transfer protein 3-like (PR14) |
