# Supplementary material for: Histological and molecular responses of Vigna angularis to Uromyces vignae infection
Source: BMC Plant Biol. 2022 Oct 14;22:489. doi: 10.1186/s12870-022-03869-2 (PMC9563176; doi:10.1186/s12870-022-03869-2)
Supplement: Supplementary file 6 — Supplementary Material 6 [file 12870_2022_3869_MOESM6_ESM.docx]

**Table S6.** DEGs associated with cell wall modification and antimicrobial compound biosynthesis at 48 hpi

| Gene_ID | logFC | q-value | Description |
| --- | --- | --- | --- |
| 108333902 | 1.01 | 1.06E-125 | Cinnamoyl-CoA reductase 1-like |
| 108322789 | 2.02 | 1.71E-06 | Peroxidase P7-like |
| 108335606 | 1.30 | 1.46E-04 | Peroxidase 5-like |
| 108322330 | 1.01 | 9.78E-12 | 3-ketoacyl-CoA synthase 1 |
| 108324448 | 1.49 | 2.81E-4 | Dihydroflavonol 4-reductase/flavanone 4-reductase-like |
