# Supplementary material for: Histological and molecular responses of Vigna angularis to Uromyces vignae infection
Source: BMC Plant Biol. 2022 Oct 14;22:489. doi: 10.1186/s12870-022-03869-2 (PMC9563176; doi:10.1186/s12870-022-03869-2)
Supplement: Supplementary file 7 — Supplementary Material 7 [file 12870_2022_3869_MOESM7_ESM.docx]

**Table S7.** Differentially expressed small heat shock proteins at 48 hpi

| Gene_ID | logFC | q-value | Description |
| --- | --- | --- | --- |
| 108337322 | 5.85 | 3.65E-170 | 17.5-kDa class-I heat shock protein-like |
| 108337748 | 4.44 | 4.69E-15 | 17.9-kDa class-II heat shock protein-like |
| 108321944 | 2.69 | 9.33E-73 | Small heat shock protein, chloroplastic-like |
| 108342410 | 2.69 | 6.90E-08 | Class-I heat shock protein-like |
| 108345352 | 2.68 | 2.86E-10 | 16.6-kDa heat shock protein-like |
| 108331196 | 1.61 | 1.86E-10 | Small heat shock protein, chloroplastic |
| 108337627 | 1.41 | 1.45E-15 | 15.7-kDa heat shock protein, peroxisomal |
| 108333576 | 1.14 | 1.73E-12 | 18.5-kDa class-I heat shock protein |
| 108331194 | 1.10 | 1.64E-31 | Heat shock 22-kDa protein, mitochondrial-like |
| 108339809 | 1.09 | 2.39E-30 | Heat shock 22-kDa protein, mitochondrial |
