# Supplementary material for: Histological and molecular responses of Vigna angularis to Uromyces vignae infection
Source: BMC Plant Biol. 2022 Oct 14;22:489. doi: 10.1186/s12870-022-03869-2 (PMC9563176; doi:10.1186/s12870-022-03869-2)
Supplement: Supplementary file 8 — Supplementary Material 8 [file 12870_2022_3869_MOESM8_ESM.docx]

**Table S8.** Primers of the genes used for qRT-PCR

| Gene_ID | Forward (5’-3’) | Reverse (5’-3’) | Amp. length |
| --- | --- | --- | --- |
| 108337322 | CTGAAGAAGGAAGAGGTGAAGG | GGTGCCATTTGTCGTTCTTG | 103 bp |
| 108342175 | GAAGTACCCTCTTTCGCTTCTC | TTCCATCTGCCGCATTTCT | 123 bp |
| 108325713 | CTTGTACGTGAGGGAGTGTTAC | GTCTACCCTTCCGTACTTCATTT | 100 bp |
| 108335120 | CACATTCTTCCGTGGGCTATTA | CTGCTGGTTCTACTGCTGATATT | 99 bp |
| 108346982 | TCTCGGGAGATTCAGAGAACT | TGAGAGGAGGAAGACAGATAAGA | 100 bp |
| 108321079 | GTACCCTCAACAAGGTGGATATG | CAAAGCACCACCAAGCAATC | 100 bp |
| 108324651 | CCTCTTCTGGGAAAGCCTAATC | TTCATTCCGAGGTTGACTTCTC | 97 bp |
| 108337247 | CTCTGGGAAACCTCAAACTCTC | GTGGAATACTGGGTTGGGTAAA | 96 bp |
| 108322622 | GTGGCAGTGTTACTGTGAAGAT | AGCATTGAGGTCAGCGATTG | 107 bp |
| 108322711 | GTGTGGGTACAGAGATGTTGAG | TACCCACGCTTGCGAAAT | 114 bp |
| 108345230 | GGTGTGTTTGGTTTGTGTAGTG | GCGCGTAAGTCCCAGTTAT | 108 bp |
| 108328346 | CCAGGAGCCAGGTTGATATT | CCTGTAGTAACTCCTCGGAAAC | 98 bp |
| 108326061 | CTTGGCCTCCATAGTTCTCTTT | TCGGGCTTTCCTTGCATAA | 96 bp |
| 108323588 | ACCCATGCAGTGCTACATAC | TCGATGTGTTAACGTCCTTCTC | 102 bp |
| 108331740 | GCAAGACATAGTGGTGGTAGAG | CAAGTGTGTAATCTTGGACTTTGG | 100 bp |
| 108332026 | TGGTGAGTGTGTCCGAAATC | TCTGAAGTAGTAGTCGGGATAGG | 125 bp |
| 108325855 | TGGGATCTACCTCCAACCTAA | CTGTGACGATGTCTCTGAAGAA | 110 bp |
| 108326022 | GCAATCACCGGATGGAATCT | AGCCGATACAGCTTTGTTCTC | 97 bp |
| 108335130 | CTTGGGTTTGGTTGCTGACA | ACCAAGTGCCAGATCAGGAA | 106 bp |
| 108324922 | CACCGTTGTTGACCCAGTTT | GCTTCCCTCTTCTGCTAGGT | 121 bp |
| 108326914 | AAAGTGGCCTCAGCTCTATTC | GCATGTTGGTGATGAGTTTGAG | 100 bp |
| 108341561 | GAGGCAGTGGTTACATAGGTTC | CCCTACCTTTGACTCGTTCTTC | 104 bp |
| 108333902 | ATGGTGCCAAGGAGAGATTG | AGGGAGAAGCAGTGTGAAAC | 108 bp |
| 108322789 | TCTGCTAGAGGGTTTGAAGTG | GAGCTGCAACAGCCAATATG | 103 bp |
| 108335606 | ACCTTGTTCGGCTCTCAATC | GTAGCCGGTAAGCACTTGTATAG | 126 bp |
| 108322330 | TGCAGTGCCGGACTTATTT | ACCAGTTGAGGGTTATGTTCTC | 106 bp |
| 108324448 | CATCGGATCATGGCTTGTAATG | GATGAAGTCAGAGGGTGAGATG | 109 bp |
| *108328964 | CTAAGGCTAATCGTGAGAA | CGTAAATAGGAACCGTGT | 165 bp |

* Represent the gene actin-3 that used as internal reference. Amp. length represent the length of PCR products for each pair of primers.
